# The effects of naturally occurring mutations on functionality of oxylipin metabolizing dehydrogenase reductase nine

**DOI:** 10.1016/j.jbc.2025.110704

**Published:** 2025-09-11

**Authors:** Samuel E. Wirth, Svetlana Pakhomova, Olga V. Belyaeva, William E. Boeglin, Alan R. Brash, Marcia E. Newcomer, Natalia Y. Kedishvili, Kirill M. Popov

**Affiliations:** 1 Department of Biochemistry and Molecular Genetics, University of Alabama at Birmingham, Birmingham, Alabama, USA; 2 Department of Biological Sciences, Louisiana State University, Baton Rouge, Louisiana, USA; 3 Department of Pharmacology, Vanderbilt University, Nashville, Tennessee, USA

**Keywords:** oxylipins, polyunsaturated fatty acids, short-chain dehydrogenase, naturally occurring mutations, protein expression, enzyme activity, protein structure, X-ray crystallography, active site

## Abstract

Recent evidence suggests that dehydrogenase reductase 9 (DHRS9) can oxidize and alter the biological activity of a diverse group of oxylipin substrates, underscoring the importance of DHRS9 in regulating various biological processes, including inflammation, cell proliferation, and tissue repair. Importantly, mutations in the DHRS9 gene resulting in amino acid substitutions S202L and D286H have been linked to an early-onset case of epilepsy; whether these mutations affect the function of DHRS9 has not been investigated. The results of this study demonstrate that both mutations cause a significant loss of DHRS9 functionality. However, in the case of the S202L variant, the loss of catalytic activity likely stems from the impaired protein folding and/or protein stability. On the other hand, the D286H DHRS9 mutant protein is relatively more stable than the S202L variant, but its *K*_m_ value for NAD^+^ (2.85 mM) is nearly 12-fold higher than that of the wild-type enzyme. The three-dimensional structure of DHRS9, solved in this study, provides insights into the functions of the S202 and D286 residues. In addition, it reveals a strikingly large substrate binding cavity, consistent with the fact that the enzyme can process oxygenated hydrocarbons with abundant rotational freedom and differing lengths (18–22 C). Considering that expression levels of DHRS9 in human tissues are highly sensitive to inflammatory conditions and the existence of naturally occurring mutations in DHRS9, the structural and functional characterization of DHRS9 reported in this study is critical for a better understanding of the role of DHRS9 in inflammatory processes.

DHRS9 is member four of the 9C family of the short-chain dehydrogenase/reductase (SDR9C4) superfamily of proteins. DHRS9 is an integral membrane protein of the endoplasmic reticulum. Based on the published literature, expression of the *DHRS9* gene is significantly altered in various pathophysiological conditions, such as rheumatoid arthritis, colorectal cancer, cicatricial alopecia, polycystic ovary syndrome, oral squamous cell carcinoma, pancreatic cancer, *etc.* (1, 2, 3, 4, 5, 6, 7, 8, 9, 10).

Until now, the importance of DHRS9 function in these diseases was attributed to its potential role as a retinol or 3*α*-hydroxysterol dehydrogenase (11, 12). However, recently, we have demonstrated that DHRS9 is a highly active oxylipin dehydrogenase (Fig. 1). It utilizes oxylipin substrates generated through 12-lipoxygenase, 15-lipoxygenase, and cytochrome P450 oxygenase pathways, and its activity towards oxylipins far exceeds its activity toward retinol or sterols (13). Among the substrates for DHRS9 are oxylipins with hydroxyl groups located at carbons C9 and C13 of octadecanoids, C12 and C15 carbons of eicosanoids, and C14 carbon of docosanoids. DHRS9 is also active toward di-hydroxylated leukotriene B_4_ and tri-hydroxylated resolvin D1 and lipoxin A_4_, although notably, it lacks activity toward 5(*S*)-HETE, lipoxin B_4,_ or the 15-hydroxyl group of prostaglandins (13).

**Figure 1:**
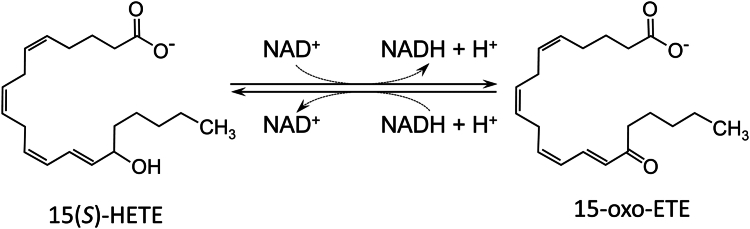
**Oxylipin dehydrogenase activity of DHRS9.** Schematic shows an interconversion of 15(*S*)-HETE and 15-oxo-ETE catalyzed by DHRS9.

Collectively, oxylipins have been implicated in the regulation of a large number of biological responses such as apoptosis, tissue repair, blood clotting, cell proliferation, blood vessel permeability, blood pressure, reproduction, diuresis, gastric secretion, smooth muscle contraction, immune actions, pain, inflammation, *etc.* (14, 15, 16, 17, 18, 19, 20, 21, 22). In particular, LTB_4_ is one of the most potent chemoattractants for neutrophils (23), whereas LXA_4_ and RvD1 were reported to act as pro-resolving mediators during inflammation (24). These observations bring up the possibility that DHRS9 may contribute to the regulation of the balance between pro-inflammatory and pro-resolving oxylipins.

Importantly, a recent study linked a case of early-onset epilepsy in a 4-year-old girl to mutations in the *DHRS9* gene (25). The patient was reported to have seizures several times a day. Initially, the seizures were drug-resistant, and were controlled by drug administration around 1 year of age. Through whole-exome sequencing of the patient’s and her parent’s nuclear DNA, two individual mutations of *DHRS9* were discovered, making the patient a compound heterozygote. Both mutations, 605C>T (S202L) and 856G>C (D286H) were found to be located in highly conserved regions of the DHRS9 protein (25). Based on "*in silico* predictive tool" analysis, the authors hypothesized that these mutations could be causative of the disease phenotype. However, whether and how these mutations impact DHRS9 enzyme activity or expression levels is currently unknown.

In this study, we used a combination of biochemical and structural approaches to characterize the effects of these naturally occurring mutations on the DHRS9 protein. Our data suggest that the mutations profoundly affect DHRS9 folding and/or stability, as well as its enzymatic activity. In addition, we report the crystal structure of DHRS9 complexed with NADH, which, for the first time, describes the active site of the enzyme, provides insights into the structure of the oxylipin-binding site, and uncovers the important structural roles of S202 and D286 amino acid residues.

## Results and discussion

### Expression of wild-type and mutant DHRS9 proteins in HEK293 cells

**Figure 2:**
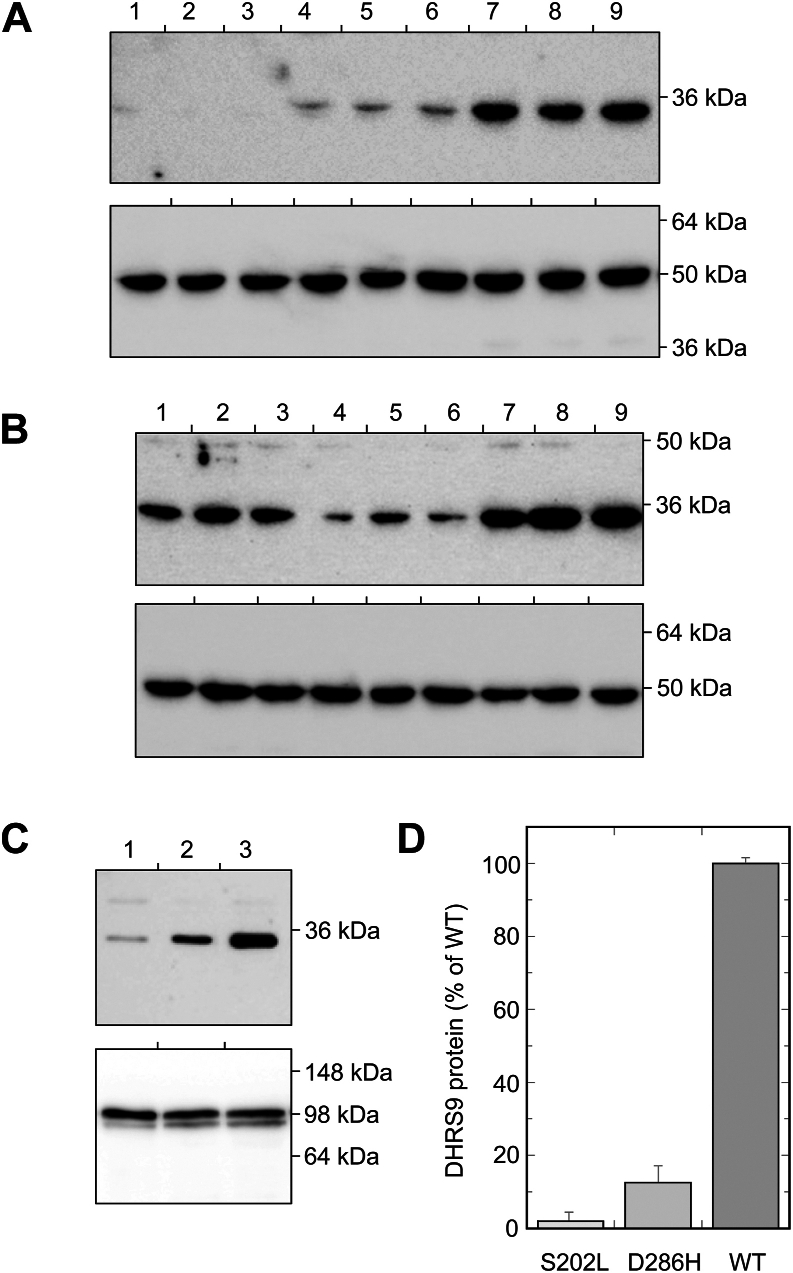
**Expression of S202L, D286H, and WT DHRS9 proteins in HEK293 cells.***A, top panel*—Western blot analysis of the whole cell extracts prepared from HEK293 cells expressing S202L (*lanes 1–3*), D286H (*lanes 4–6*), and WT DHRS9 (*lanes 7–9*); *bottom panel*—same samples probed with β-actin antibodies as loading control. *B, top panel* – Western blot analysis of the whole cell extracts prepared from HEK293 cells co-expressing either S202L with WT DHRS9 (lanes 1–3), S202L with D286H DHRS9 (lanes 4–6), or D286H with WT DHRS9 (lanes 7–9); *bottom panel*—the same samples probed with β-actin antibodies as loading control. *C, top panel*—Western blot analysis of microsomes isolated from HEK293 cells expressing individual S202L, D286H, and WT DHRS9 proteins; *bottom panel*—the same samples probed with calnexin antibodies as loading control. *D*, relative amounts of S202L, D286H, and WT DHRS9 proteins in microsomes isolated from HEK293 cells.

To initiate the characterization of the effects of the naturally occurring mutations on DHRS9 protein, plasmids carrying cDNAs for the wild-type (WT) and mutant forms of DHRS9 in-frame with the C-terminal FLAG tag were transiently expressed in HEK293 cells as described under Experimental Procedures. Twenty-four hours post-transfection, the cells were harvested, and the levels of DHRS9 proteins were analyzed by Western blotting of the whole-cell extracts using FLAG antibodies. As shown in Figure 2*A*, both mutations, *i.e*., S202L and D286H, drastically decreased the levels of the corresponding recombinant proteins. The levels of S202L DHRS9 protein were on the border of detection sensitivity.

To determine whether the reduced protein levels of DHRS9 mutants were caused by destabilization of the mRNA, we performed Northern blot analysis of the corresponding mRNA levels. Northern blot analysis showed that the mRNAs encoding the mutant variants were expressed at levels similar to the WT mRNA (Fig. S1), suggesting that the S202L and D286H mutations are likely to affect the accumulation of functional enzyme.

As described below, our data show that DHRS9 exists as a dimer. This brings up the possibility that the mutant forms of DHRS9 could have destabilizing effects on the WT enzyme and on each other when they form mixed heterodimers. To examine this possibility, we carried out transient expression experiments, in which HEK293 cells were co-transfected with expression plasmids for WT DHRS9 and one of the mutants, or the two mutants together. As illustrated in Figure 2*B*, the co-transfection experiments clearly demonstrated that the mutant forms of DHRS9 do not exert an appreciable destabilizing effect on the WT protein or on each other. These observations are consistent with the interpretation that the effects of mutations largely come about because of interference with DHRS9 folding.

Data from this and other laboratories indicate that DHRS9 is an integral membrane protein associated with microsomal membranes (11, 12, 13). To determine whether the mutations caused miss-targeting of the DHRS9 protein, we examined the subcellular localization of the mutant variants. Western blot analysis showed that the mutant forms of DHRS9 were associated with the microsomal fractions similarly to the WT protein (Fig. 2*D*). However, as would be expected from the experiments with whole-cell extracts, the protein levels of DHRS9 mutant proteins in microsomes were greatly diminished relative to the levels of the WT protein. Based on the quantification of several independent transfection experiments, the levels of S202L DHRS9 were almost 40-fold lower than those of the WT protein, while the levels of D286H mutant were approximately 7-8-fold lower compared to the WT DHRS9 (Fig. 2*D*).

### Characterization of catalytic activities of wild-type and mutant DHRS9 proteins

**Figure 3:**
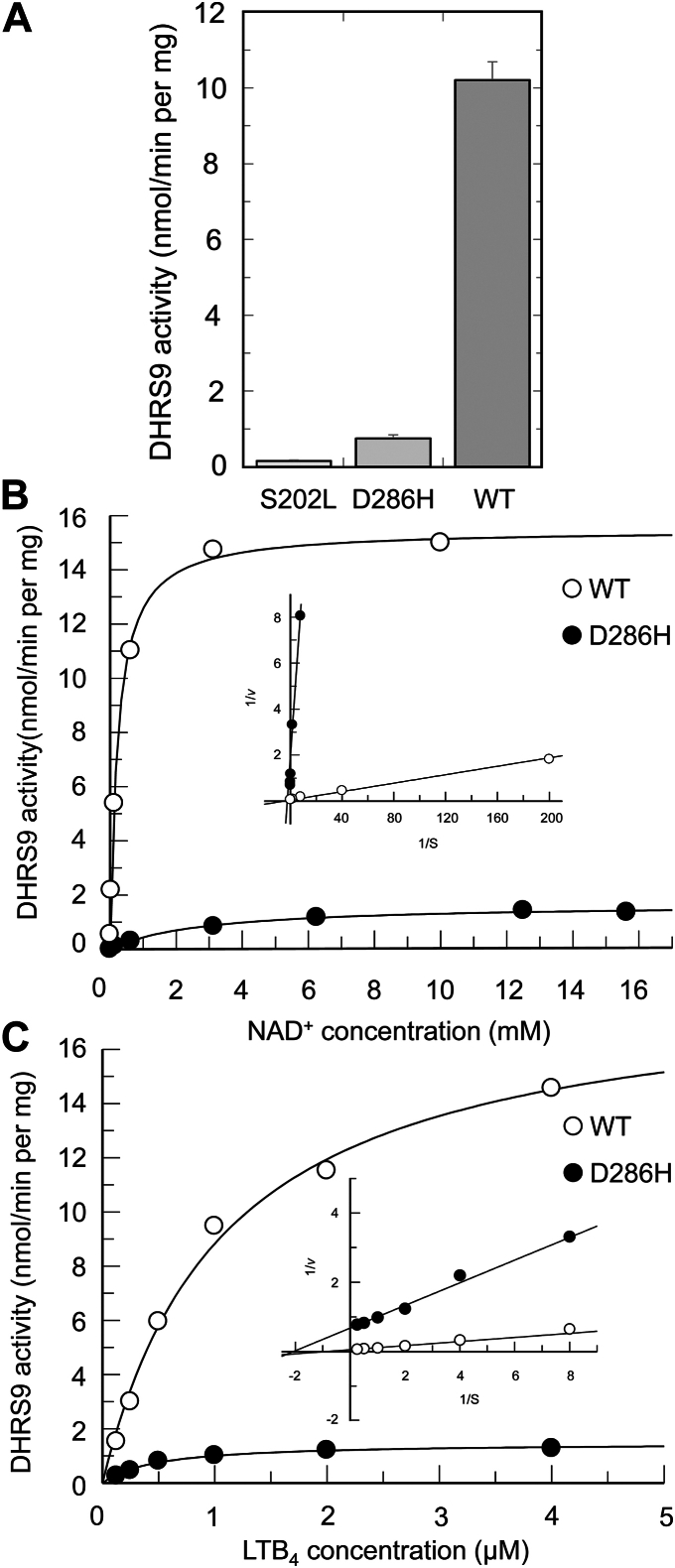
**Catalytic activity of S202L, D286H, and WT DHRS9 in microsomes isolated from HEK293 cells.***A*, activities of S202L, D286H, and WT DHRS9 proteins determined using 5 μM 15(*S*)-HETE and 4 mM NAD^+^. *B*, kinetics of D286H and WT DHRS9 with respect to nucleotide co-factor NAD^+^ (*inset*, Lineweaver-Burk plot). *C*, kinetics of D286H and WT DHRS9 with respect to oxylipin substrate LTB_4_ (*inset*, Lineweaver-Burk plot).

The presence of mutant DHRS9 proteins in the microsomal fractions isolated from HEK293 cells indicated that they were correctly targeted to the endoplasmic reticulum membranes and, therefore, could retain some catalytic activity. To examine this possibility, we determined DHRS9 activity in microsomes isolated from cells expressing DHRS9 mutant and WT proteins under standard conditions that were previously developed for the analysis of WT DHRS9 (13). As illustrated in Figure 3*A*, microsomes containing S202L and D286H variants of DHRS9 displayed greatly decreased oxylipin dehydrogenase activities, as would be expected from the Western blot data. In fact, the oxylipin dehydrogenase activity in microsomes expressing S202L DHRS9 did not show statistically significant increase over the blank values obtained with microsomes isolated from HEK293 transfected with empty vector. Our attempt to improve the sensitivity of the assay by increasing the incubation times or the amounts of total protein in the assay failed to identify statistically significant activity in microsomes expressing S202L DHRS9 protein, which is consistent with interpretation that microsomal S202L DHRS9 is, at least partially, unfolded and lacks an appreciable catalytic activity. In contrast, microsomes expressing D286H DHRS9 displayed statistically significant oxylipin dehydrogenase activity (Fig. 3*A*). As expected, this activity was significantly lower than that observed in microsomes expressing WT enzyme (approximately 14-fold). However, it could be readily measured and analyzed.

This allowed us to characterize the kinetics of oxylipin dehydrogenase activity catalyzed by D286H DHRS9 enzyme and compare it to the kinetics of the WT enzyme. As shown in Figure 3*B*, D286H DHRS9 enzyme followed Michaelis-Menten kinetics with respect to the nucleotide co-factor NAD^+^. Importantly, the D286H DHRS9 variant displayed a greatly decreased affinity for NAD^+^ relative to the WT enzyme (the apparent *K*_m_ value 2.85 ± 0.45 mM *versus* 0.23 ± 0.02 mM, respectively). Under the described conditions, the apparent *V*_max_ value displayed by the D286H mutant was approximately 9-fold lower than that of the WT DHRS9, but this outcome would be expected based on the disparities in protein expression levels (Fig. 2, *C* and *D*).

To characterize D286H DHRS9 kinetics with oxylipin substrate, we used di-hydroxylated substrate leukotriene B_4_ or LTB_4_ (Fig. 3*C*). With LTB_4_ as a substrate, the activity of D286H variant followed Michaelis-Menten kinetics and displayed an apparent *K*_m_ value that was slightly lower than the apparent *K*_m_ value for the WT enzyme (0.50 ± 0.06 μM *versus* 1.0 ± 0.14 μM, respectively). As would be expected, the apparent *V*_max_ value displayed by D286H DHRS9 was significantly lower than that of the WT enzyme (1.5 ± 0.06 nmol/min per mg *versus* 18.5 ± 1.0 nmol/min per mg).

Taken together, the results of these experiments strongly suggested that both mutations caused significant loss of DHRS9 functionality. However, these outcomes occurred as a result of somewhat different molecular defects. In the case of the S202L variant, the loss of catalytic activity came about as a result of a significant decrease in total DHRS9 protein caused by problems with protein folding and/or protein stability. Even the trace amounts of S202L DHRS9 targeted to microsomes displayed little if any catalytic activity, indicating that a properly targeted enzyme may be partially unfolded. D286H DHRS9 also displayed a greatly diminished catalytic activity. However, the causes of these changes appeared to be more complicated. Similar to the S202L variant, the large decrease in the activity of D286H DHRS9 enzyme occurred as a result of a decrease in the total amount of protein caused by problems with protein folding and/or protein stability, but in contrast to the S202L variant, the D286H DHRS9 protein targeted to microsomes appeared to be catalytically active. When corrected for the protein levels, its activity was only somewhat lower than the activity of the WT enzyme. However, our kinetic studies demonstrated that the D286H mutant displayed a major catalytic defect; its apparent *K*_m_ value for the nucleotide cofactor was as high as 2.85 mM. In mammals, the cellular concentration of NAD^+^ ranges from 200 μM to 500 μM, indicating that *in vivo* D286H enzyme must have a greatly diminished activity compared to the WT counterpart because of its poor affinity for NAD^+^. Based on these considerations, it appears to be reasonable to assume that the D286H variant should display a very low activity with oxylipin substrates *in vivo*.

### Three-dimensional structure of DHRS9

To get additional insights into the functions of S202 and D286 amino residues, we determined the three-dimensional structure of DHRS9. To crystallize DHRS9, we created a water-soluble form of the mouse enzyme by removing the N-terminal amino acids forming the transmembrane segment (Fig. S2*A*). The resulting enzyme was isolated using metal-affinity chromatography. Purified protein showed a single band of expected molecular size of approximately 36 kDa on SDS/PAGE (Fig. S2*B*). In solution, the water-soluble form of DHRS9 formed stable dimers (Fig. S3), which is characteristic of many SDRs (26). As shown in Fig. S4, the purified protein was catalytically active and could utilize a diverse group of mono, di-, and tri-hydroxylated oxylipin substrates.

As described under Experimental Procedures, the highly purified water-soluble form of DHRS9 was crystallized in complex with NADH. As shown in Table 1, crystals belonged to the monoclinic space group *C2* with *a* = 143.1 Å, *b* = 74.21, *c* = 102.8 Å, *β* = 133.1° with a dimer in the asymmetric unit. Twenty-four residues coding for the added His_10_-tag sequence along with residues 17 to 20 (chain A) and 17 to 21 (chain B) of DHRS9 protein could not be located. Consequently, the final model of DHRS9/NADH complex consists of protein residues 21 to 319 (chain A), 22 to 319 (chain B), two NADH molecules and 32 water molecules. The structure was refined to 2.00 Å resolution with R of 20.15% and an R_free_ of 23.84% (Table 1). It contained seven β strands forming a parallel β sheet and 12 α helices (Table S1 and Fig. S5).

**Table 1:** Data collection and refinement statistics

	DHRS9/NADH complex
Wavelength (Å)	0.9791
Resolution (Å)	50-2.00 (2.11-2.00)
Temperature (K)	100
Space group	*C2*
Cell dimensions	
*a* (Å)	143.1
*b* (Å)	74.21
*c* (Å)	102.8
*β* (°)	133.1
Number of molecules per asymmetric unit	2
No. of unique reflections	52,809
R_*pim*_^a^^,^^b^ (%)	3.3 (42.6)
Completeness (%)	99.3 (99.1)
Redundancy	4.5 (4.5)
*I/σ(I)*	10.5 (2.0)
CC(1/2)	0.999 (0.809)
Refinement statistics	
Resolution range	40–2.00
No. of reflections used in refinement	49,599
*R*/*R*_*free*_^c^ (%)	20.15/23.84
Number of refined atoms	
Protein	4599
Heterogen atoms	88
Water	32
Average B-factors (Å^b^)	
Protein	76.2
Nucleotide	72.1
Water	65.5
R.m.s. deviations	
Bonds (Å)	0.007
Angles (°)	1.426
Ramachandran plot (%)	
Favored	95.28
Disallowed	0.34

a Values in parentheses are for the highest-resolution shell.

b R_pim_ is a redundancy-independent measure of the quality of intensity measurements. R_pim_ = ∑_hkl_ (1/(n −1))^1/2^ ∑_i_ |I_hkl,i_ - <I_hkl_>|/∑_hkl_ ∑_i_ I_hkl,i_, where I_hkl,i_ is the scaled intensity of the measurement of reflection h, k, l, <I_hkl_> is the average intensity for that reflection, and n is the redundancy.

c R = ∑|| F_o_ | - | F_c_ ||/∑|F_o_|, where F_o_ and F_c_ are the observed and calculated structure factors amplitudes. R_free_ is calculated using 5.1% of reflections omitted from the refinement for the DHRS9/NADH structure.

**Figure 4:**
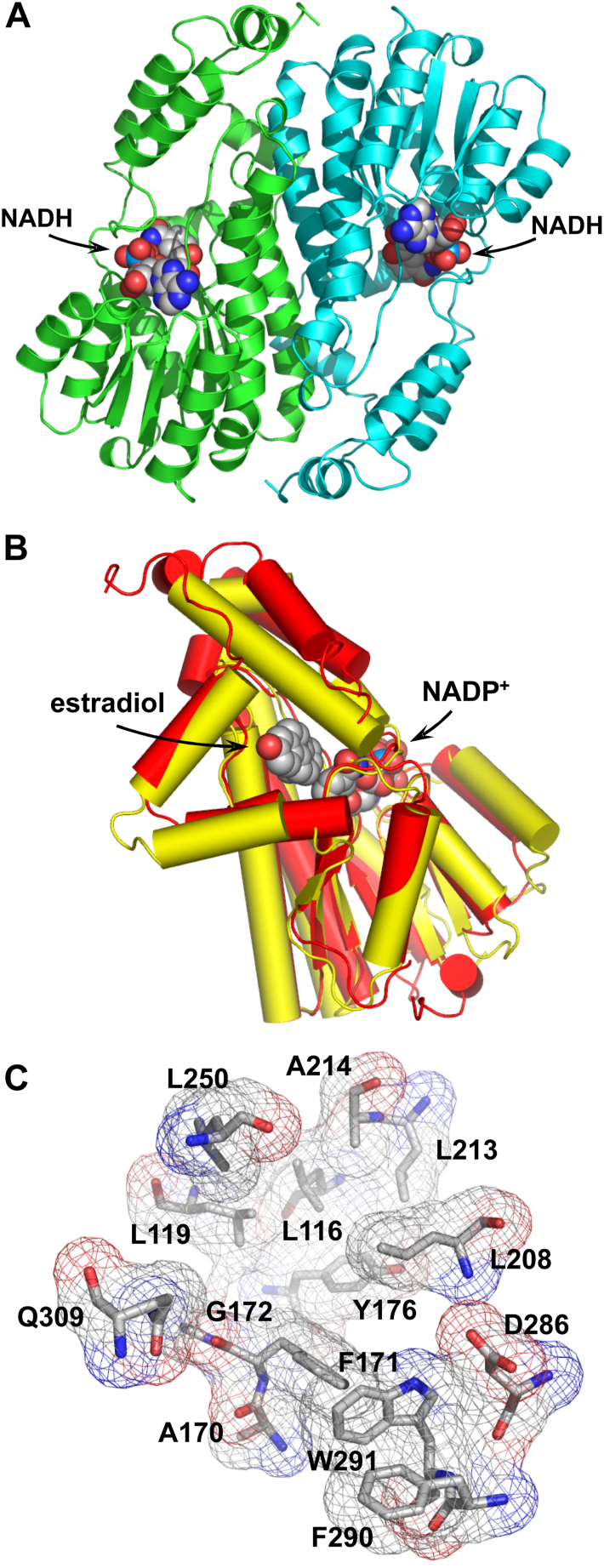
**The three-dimensional structure of DHRS9/NADH complex.***A*, ribbon representation of DHRS9 dimer with NADH molecules bound to the nucleotide-binding pockets (*chain A* in *green*, *chain B* in *blue*). The structure is viewed along the dimerization interface. The bound NADH molecules are shown as space-filling models. *B*, the structure of DHRS9 without bound NADH superimposed with the structure of 17*β*-HSD1 (PDB accession number 1A27). The structure of DHRS9 is colored in *red*. The structure of 17*β*-HSD1 is colored in *yellow*. The structures of estradiol and NADP+ bound to 17*β*-HSD1 are shown as space-filling models. The structural alignment of DHRS9 and 17*β*-HSD1 sequences can be found in Fig. S6. *C*, the structure of the oxylipin-binding cavity of DHRS9. The amino acid residues contributing to the structure of the oxylipin-binding cavity are shown as stick models. Superimposed are the molecular surfaces of amino acid residues forming the oxylipin-binding cavity (surfaces are shown as mesh representation). Carbon is shown in *grey*, oxygen in *red*, nitrogen in *blue*.

The overall fold determined for DHRS9/NADH complex is shown in Figure 4*A*. Its central parallel β-sheet (strands β1-β7) was surrounded on each side by two groups of α-helices on one side and on the other side (helices α1, α2, α9 and helices α3, α4, α5, respectively). In addition, it contained a helical subdomain at the C-terminal end of the beta sheet. This α/β type structure that includes the so-called Rossmann fold is often encountered in dinucleotide-binding domains (27). The overall fold of DHRS9 appeared to be characteristic of the short-chain dehydrogenase/reductase superfamily (26). It also incorporated all the conserved consensus sequences, such as the amino-terminal ThrGlyXXXGlyXGly cofactor binding motif (27); a conserved AsnAsnAlaGly sequence, which is important for stabilization of the central *β*-sheet (28, 29); as well as the catalytic tetrad Asn-Ser-Tyr-Lys (27).

A very well-defined electron density was observed for the NADH molecules in the DHRS9/NADH complex. The cofactor molecule was bound in the extended conformation with the nicotinamide moiety in *syn* conformation and the adenine ring in the *anti* conformation. The NADH-binding cavity of DHRS9 was largely shaped by amino acids Gly36, Asp38, Thr39, Phe41, Asp83, Val84, Asn112, Lys180, Tyr176, Phe209, Thr211, and Glu212. DHRS9-bound NADH was stabilized through multiple hydrogen bonding interactions with amino acids forming the NADH-binding cavity of the enzyme. The nicotinamide of NADH was stabilized through the hydrogen bonding interactions with Phe209 and Thr211.

Previous studies established an important role of the catalytic tetrad (Asn-Ser-Tyr-Lys) in promoting dehydrogenase reaction (29). It is generally believed that Tyr functions as the catalytic base (30). Ser stabilizes the substrate, and Lys lowers the pKa of Tyr residue to facilitate the proton transfer (29). In DHRS9, the catalytic tetrad was represented by residues Asn137, Ser164, Tyr176, and Lys180 (for more details, see Fig. S6). Their side chains could be clearly identified in the vicinity of the nicotinamide moiety of NADH and retained the geometry characteristic of the catalytic tetrad found in other short-chain dehydrogenases/reductases (29).

It is generally believed that dimerization of short-chain dehydrogenases/reductases is crucial for their activity (26, 31, 32). Based on PISA server calculations (33), in the case of DHRS9, the dimerization interface buried a total of 7400 Å^2^ of solvent accessible area. Dimerization was mediated by the interactions between two long helical motifs (helices α4 and α5) coming from each monomer. They formed a helical bundle with an average surface interaction area of 2371 Å^2^ (15.6%) per monomer. The interface was largely stabilized by strong hydrophobic and aromatic interactions (Val127, Tyr130, Ile134, Leu138, Phe139, Ile142, Leu146), as well as a salt bridge between Arg131 and Glu135. This arrangement helps to position two catalytic residues, Tyr176 and Lys171, located on the interior side of the α5 helix toward the nicotinamide ring of nucleotide cofactor and the OH group of oxylipin substrate, thus allowing the oxidation reaction to occur.

In short-chain dehydrogenases/reductases, the substrate-binding site is usually located toward the carboxyl end (34, 35). Analysis of the surface topography of DHRS9/NADH complex using the CASTp server (36) identified only one significant solvent-accessible cavity located at the carboxyl end of DHRS9. As illustrated in Figure 4*B*, it appears that this cavity is superimposable with the estradiol-binding site of 17*β*-HSD1 (35) and, therefore, it is highly likely that it represents the site of oxylipin binding (for sequence alignment see Fig. S7). This putative oxylipin-binding site was largely shaped by the hydrophobic amino acids such as Leu116, Leu119, Ala170, Phe171, Tyr176, Leu208, Leu213, Ala214, Leu250, Phe290, and Trp291 (Fig. 4*C*), which are presumably necessary to accommodate the large, highly hydrophobic polycarbon chains of various oxylipin substrates. In general, this oxylipin-binding cavity appeared to be rather large (a solvent accessible volume of 974 Å^3^ and surface area of 1293 Å^2^; these values also include the cofactor binding site). It significantly exceeded the size of the estradiol-binding cavity in 17*β*-HSD1 (425 Å^3^ and 732 Å^2^, respectively). The larger cavity volume is likely necessary to accommodate the distinct orientations of various highly flexible oxylipin substrates of 18 to 22 carbons in length.

**Figure 5:**
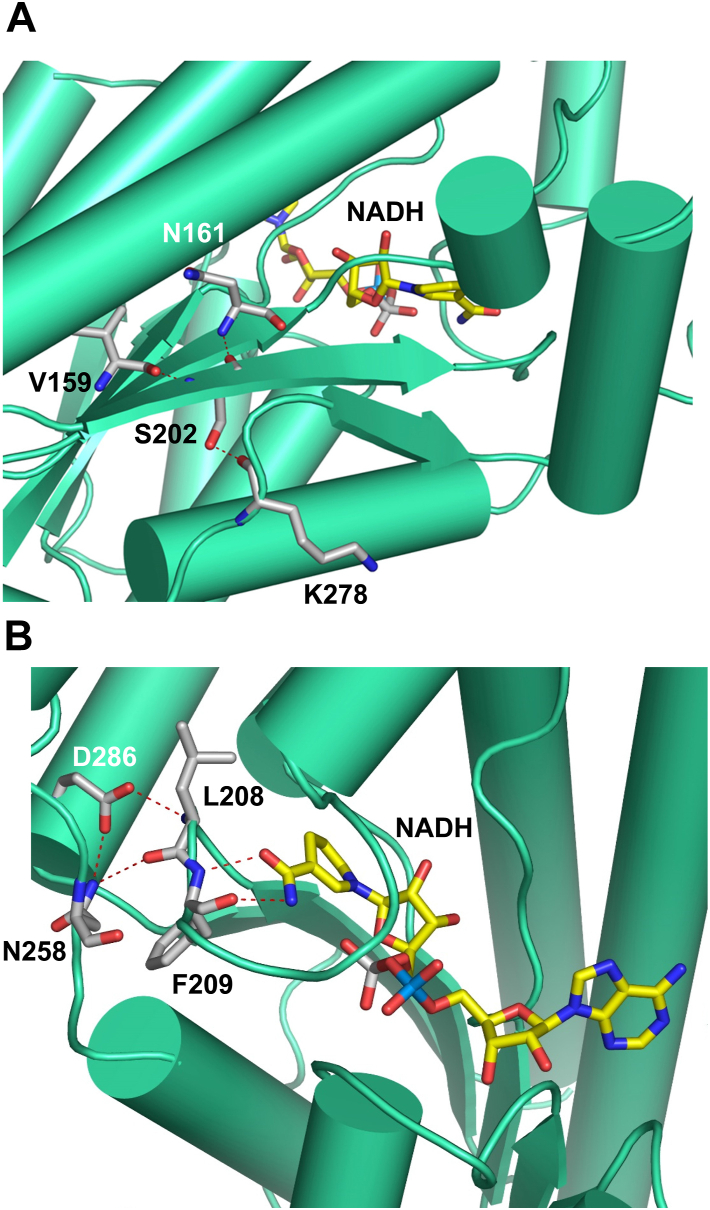
**The structural roles of S202 and D286 amino acid residues.***A*, the structural role of the S202 residue. *B*, the structural role of D286 residue. Interacting amino acid residues are shown as stick models. Carbon is shown in *grey*, oxygen in *red*, nitrogen in *blue*. Hydrogen bonds are indicated by the *red dashed lines*. The bound NADH molecules are shown as stick models. Color coding is as follows: carbon *yellow*, oxygen *red*, nitrogen *blue*, phosphate *grey* or *light blue*.

Considering that amino acid sequences of the mouse and human enzymes are 87% identical (Fig. S8), determination of three-dimensional structure of mouse DHRS9 allowed us to further examine the functional significance of S202 and D286 amino acid residues as it pertains to the human enzyme. As illustrated in Figure 5*A*, S202 is an integral part of β6 strand located at the “heart” of α/β core characteristic of dehydrogenase/reductase enzymes. It helps to stabilize the α/β core through the extended hydrogen bonding network. Interactions with V159 and N161 located in β5 strand help to align and stabilize strands β5 and β6 relative to each other. In addition, the hydroxyl group of S202 side chain forms hydrogen bond with K278 (Fig. 5*A*). K278, in turn, is adjacent to the strand β7. Consequently, its interaction with S202 helps to bring strand β7 to the central β sheet and properly align it. The substitution of S202 with leucine residue will prevent this interaction, thereby destabilizing α/β core of DHRS9. Importantly, strand β7 is immediately adjacent to the helices α10 and α11, which form the part of the mouth of oxylipin-binding cavity. The location of S202 in the crystal structure is consistent with the hypothesis that the S202L mutation interferes with DHRS9 folding by destabilizing the α/β core and can explain our biochemical data showing the extremely low levels of S202L DHRS9 expression and its lack of enzyme activity (Figs. 2 and 3*A*).

D286 residue is an integral part of the α10 helix, which, along with the adjacent α11 helix, participates in the formation of the mouth of the oxylipin-binding cavity. Similar to S202, D286 forms an extended hydrogen bonding network helping to stabilize the loops within the active site of DHRS9 (Fig. 5*B*). Elimination of this hydrogen bonding should interfere with the folding of the active site, which potentially might destabilize the overall structure of the D286H DHRS9 mutant. The low expression levels of D286H DHRS9 protein that were observed in HEK293 cells (Fig. 2) are consistent with this hypothesis. As illustrated in Figure 5*B*, D286 forms hydrogen bonds with L208 and N258. N258, in turn, is also hydrogen-bonded to L208, thereby stabilizing the position of the protein loop around the nicotinamide moiety of the nucleotide cofactor. Importantly, L208 is immediately adjacent to F209 (Fig. 5*B*). F209, as discussed above, forms hydrogen bonds with the nicotine amide moiety of nucleotide cofactor, helping to bind and position it for the catalysis of the dehydrogenase reaction. Therefore, the disruption of this hydrogen bonding network should affect the enzyme’s ability to utilize the nucleotide cofactor. In agreement with these observations, we saw a great decrease in the affinity of D286H DHRS9 for NAD^+^ (Fig. 3*B*), which, along with poor expression, likely accounts for the low activity of the mutant enzyme *in vivo*.

Taken together, our biochemical and structural data clearly indicate that S202L and D286H mutations should compromise DHRS9 activity *in vivo*. Additional experiments will be required to establish how the compromised activity of DHRS9 can lead to the pathogenesis of diseases such as epilepsy. In this context, our earlier studies (13) demonstrated that DHRS9 can metabolize and inactivate a wide range of mono-, di-, and tri-hydroxylated oxylipins having pro-inflammatory and pro-resolution activities. Based on the kinetics of the DHRS9-catalyzed reaction, the enzyme's activity should move the balance from the pro-inflammatory to pro-resolving oxylipins. Therefore, elimination of DHRS9 should cause dysregulation of the oxylipin profile by favoring the accumulation of pro-inflammatory oxylipins and promoting inflammation as a result. In this context, epilepsy has been described as an inflammatory disease for many years now (37, 38, 39). Moreover, certain cases of epilepsy have even been effectively treated with anti-inflammatory drugs (40, 41). Our data shows that DHRS9 mutants S202L and D286H are biologically inactive, suggesting that DHRS9 deficiency may serve as a causal link or exacerbate the pathophysiology of certain types of epilepsy.

## Experimental procedures

### Reagents

Oxylipins were purchased from Cayman Chemical.

### Expression of S202L, D286H, and wild-type DHRS9 in HEK293 cells

There are multiple transcripts reported for *DHRS9* gene (GeneID:10170), and multiple deduced protein isoforms. NM_001289763 referenced in (25) corresponds to the transcript form five encoding a 379 amino acid-long protein isoform 2, which contains additional 60 N-terminal amino acids in comparison to the 319 amino acid-long isoform 1. In this manuscript, we used the numbering corresponding to isoform 1 (https://www.ncbi.nlm.nih.gov/nuccore/NM_199204.2. NP_954674), also known as RDH-like (RDHL), which was shown to match the natively occurring protein [Fig. 1*D* in ref. 42]. Human DHRS9 cDNA from the previously described expression construct (42) was PCR-amplified using primers 5′-TTT GGA TCC ATG CTC TTT TGG GTG CTA GGC-3′ and 5′-TTT CTC GAG CAC TGC CTT GGG ATT AGC CAG -3′ restriction sites underlined*)* and cloned into *Bam*HI*-Xho*I sites of pCMV-Tag4a vector (Stratagene) in-frame with the C-terminal FLAG tag. S202L and D286H mutations were introduced using site-directed mutagenesis with FLAG-tagged DHRS9/pCMV-Tag4a as a template, Q5 High-Fidelity DNA polymerase (New England Biolabs), and primers 5′-GCA TTG AAC CAG GAT TGT TCA-3′ (forward) and 5′-ATA AGA CGT GCA CAC CAA AAG C-3′ (reverse, substitution underlined) for S202L mutant, and 5′-GCC GCT GGA AAA CAT GCC AAA-3′ (forward, substitution underlined) and 5′-ATA ATG AGT CTT AGG GAA GAG ACT-3′ (reverse) for D286 mutant. HEK293 cells were cultured in minimal essential medium containing 10% horse serum and penicillin/streptomycin at 37 °C with 5% CO_2_ (genotyped and *mycoplasma* tested HEK293 cells were obtained from American Type Culture Collection). For DHRS9 overexpression, HEK293 cells were plated in 35-mm dishes and transiently transfected with sequence-verified DHRS9 constructs or equal amount of an empty vector using Lipofectamine 3000 (Life Technologies) according to the manufacturer's protocol. Twenty-four hours after transfection, cells were collected, rinsed with PBS, and aliquots of cell suspensions were taken for protein quantification, Western blot analysis, and isolation of microsomes. Double-transfection experiments were conducted in a similar fashion.

### Subcellular fractionation of HEK293 cells

Cells were homogenized on ice with a Dounce homogenizer in PBS with 0.25 M sucrose, 1 mM EDTA and protease inhibitors (2 μg/ml of aprotinin, 2 μg/ml of leupeptin, 1 μg/ml of pepstatin-A and 5 mM benzamidine. Homogenates were centrifuged at 3000*g* for 10 min to obtain postnuclear fraction. Postnuclear fractions were centrifuged at 10,000*g* to pellet crude mitochondrial fraction. The 10,000*g* supernatants were centrifuged at 40,000 rpm in SW55Ti Beckman rotor for 1 h. Microsomal and mitochondrial pellets were resuspended in 90 mM potassium phosphate, pH 7.4, 40 mM potassium chloride (reaction buffer), supplemented with 1 mM dithiothreitol and 20% glycerol, and stored in small aliquots at −80 °C. Protein concentrations were determined using BioRad DC Protein Assay with BSA as a standard.

### Western blot

For Western blot analysis, the total cell homogenates or microsomal protein fractions were separated in 12% SDS-PAGE, transferred to Immobilon-P PVDF membrane (Millipore-Sigma) and blocked in 4% BSA in TBST buffer. Blots were incubated overnight at 4 °C in blocking buffer with *anti-*FLAG rabbit polyclonal antibody (Millipore-Sigma, F7425) at 1:2500. Secondary horseradish peroxidase-conjugated anti-rabbit antibodies (#7074, Cell Signaling Technology, Danvers, MA) were used at a 1:10,000 dilution. Immunoreactive bands were detected using the Immobilon Forte Western HRP Substrate (Millipore-Sigma). Imaging was performed using the ChemiDoc MP Imaging System (Bio-Rad). Blots were quantified with UN-SCAN-IT software (Silk Scientific Inc.). Loading of the samples for the whole cell extracts was evaluated using β-actin antibodies (Millipore, A1978–100UL) at a 1:4000 dilution. Loading of the microsomal fractions was analyzed using calnexin antibodies (StressGen, spa-865) at a 1:3000 dilution.

### Determination of DHRS9 activity

The activities of microsomal and water-soluble DHRS9 were determined following protocols described previously (13). Briefly, measurements were performed in 90 mM potassium phosphate, 40 mM KCl, pH 7.3 at 37 °C in glass siliconized tubes. Oxylipin substrates were prepared for reactions in a stock solution that varied in concentration based on the substrate, but always with an ethanol/methanol final concentration of ≤1%. The stock solution was made in the previously described buffer conditions; in addition, fatty acid-free BSA at an equimolar concentration was used as a solubilizing agent for all substrates. Reactions were carried out either in 0.5 ml or 1 ml volumes and were started by the addition of the cofactor. The 0.5-ml reactions were stopped with either the addition of 0.5 ml ethyl acetate and 2 ml of hexane (LTB_4_, 9(*S*)-HODE, 12(*S*)-HETE, 13(*S*)-HODE, 15(*S*)-HETE) or 2.5 ml methylene chloride (RvD1, LXA_4_). One-ml reactions were stopped with 2× volumes of the previously mentioned solvents. Activity measurements for transformation experiments were determined in triplicate. Transformation experiments were repeated at least 5 to 6 times.

For kinetic studies, the apparent *K*_m_ and *V*_max_ values of D286H and WT DHRS9 for NAD^+^ were determined using fixed concentrations of LTB_4_ of 5 and 10 μM, respectively, whereas the apparent *K*_m_ and *V*_max_ values of D286H and WT DHRS9 for LTB_4_ were determined using fixed concentrations of NAD^+^ of 28.5 and 2.3 mM. Initial velocities (nmol of product formed per min per mg of total protein) were obtained by linear regression. The amount of product formed was less than 15% within the 15-min reaction time and was linearly proportional to the amount of protein added. Each kinetic experiment was repeated at least three times. Controls without added cofactor were included with each experiment. Kinetic data were analyzed, fitted, and plotted using GraFit Version five software (Erithacus Software Limited). Kinetics for soluble form of mouse DHRS9 were analyzed in a similar fashion.

### Northern blot analysis

Northern blot analysis was performed using 20 μg of total RNA from HEK293 cells expressing S202L, D286H, WT DHRS9, or control vector constructs. Total RNA was isolated using TRIzol reagent according to the manufacturer’s instructions. The total RNA isolate was then separated on a 1.2% agarose formaldehyde gel and transferred to a nylon membrane *via* upward capillary transfer. Blots were hybridized with digoxigenin-11-dUTP labelled 960 bp–long probe derived from the full-length cDNA of DHRS9. The probe labeling and band detection were performed using the DIG-High Prime DNA Labeling and Detection Starter Kit I from Roche (Cat. 11745832910) following the manufacturer's protocol. Imaging was performed using the ChemiDoc MP Imaging System (Bio-Rad).

### Expression and purification of water-soluble DHRS9

As reported previously (11, 13), DHRS9 is an integral membrane protein. Analysis of the DHRS9 sequence using transmembrane topology prediction software DeepTMHMM (43) identified a single amino-terminal segment, which is presumably responsible for the incorporation of the DHRS9 protein into the endoplasmic reticulum membrane (Table S1). Thus, to create a soluble form of DHRS9, we constructed a partial cDNA lacking the sequence coding for the first 16 amino acids forming the transmembrane segment. The coding sequence of mouse DHRS9, including the stop codon, was generated by RT-PCR using templates prepared from skin. The resulting cDNA was subcloned between *BamH*I and *EcoR*I sites of the pCMV-Tag4a vector (Stratagene). cDNA integrity was verified by sequencing (13). To generate a soluble form of DHRS9, the unique *Nde*I site flanking sequence coding for the amino-terminal transmembrane segment was introduced into cDNA coding for the full-length DHRS9. *NdeI*/*BamH*I cDNA fragment coding for a soluble form of DHRS9 was subcloned between *Nde*I and *BamH*I sites of pET-19b expression vector obtained from Novagen (pDHRS9delta vector). This created a chimeric construct carrying the His_10_-Tag at the amino-terminal end of the truncated DHRS9 protein, thus allowing for a rapid purification of the resulting soluble form of DHRS9 protein using metal-affinity chromatography.

To establish the expression cell line, competent BL21(DE3) cells obtained from Millipore/Sigma were transformed with the pDHRS9delta expression vector. Transformants were selected on LB agar containing 100 μg/ml ampicillin. Several colonies displaying ampicillin resistance were tested for their ability to express soluble DHRS9 and were used to prepare glycerol stocks. To express DHRS9 for purification, three 50 ml cultures made of Terrific Broth with 100 μg/ml ampicillin were inoculated with BL21(DE3) cells transformed with the pDHRS9delta vector. Cultures were incubated at 37 °C with constant shaking at 250 rpm until they reached an optical density of 0.6 at 600 nm. At this point, they were transferred to 28 °C and incubation was continued for another 18 *h*. After 18 *h*, cells were harvested by centrifugation at 5000*g* for 20 min at 4 °C. The resulting pellet was washed in 20 mL of 50 mM Tris, pH 7.9. Cells were recovered again by centrifugation and treated for 30 min at 4 °C with 10 ml Tris/Saline/EDTA buffer containing 1 mg/ml egg yolk lysozyme. The lysozyme-treated cells were recovered by centrifugation and stored at −70 °C for at least 1 *h*.

To purify DHRS9, the pellet of lysozyme-treated cells was resuspended in 9 ml of ice-cold 50 mM sodium phosphate buffer (pH 8.0) containing 300 mM NaCl, 10 mM imidazole, 10% glycerol, 1 mM PMSF, 1 μl/ml Benzonase (Novagen), one pellet of Roche protease inhibitor mix (Mini, EDTA-free), 1 ml of 10 × BugBuster (Novagen), and 1% Elugent detergent. Resuspended cells were disrupted by sonication (three times for 20 s) with 1 min intervals for cooling on ice. Sonicated cells were incubated for 25 min at room temperature. The resulting extract was clarified by centrifugation at 16,000*g* for 30 min at 4 °C. The supernatant was loaded onto a 1 ml Ni NTA (Qiagen) column at 4 °C. After loading, the column was washed with 10 ml 50 mM sodium phosphate, pH 8.0, containing 300 mM NaCl, 20 mM imidazole, 50 mM Elugent, and 20% glycerol. Bound DHRS9 was eluted using six consecutive washes (600 μl each) of the same buffer mixture adjusted to 500 mM imidazole. The purified enzyme was analyzed by SDS/PAGE. The gels were stained using Coomassie G-250. The gel images were generated using Bio-Rad Gel Doc EZ imager (software version Image Lab ver.6.1.0). DHRS9 containing fractions were pooled, desalted on a PD-10 column (GE Healthcare) equilibrated with 50 mM sodium phosphate, 1 M NaCl, 50 mM Elugent and 20% glycerol, and stored at −70 °C.

### Protein crystallization

To generate crystals of DHRS9/NADH complex, DHRS9 protein was preincubated with 1.5 mM NADH prior to crystallization. Crystals were obtained using the hanging drop vapor-diffusion method by mixing equal volumes of protein (5.5 mg/ml concentration in 25 mM Na phosphate, pH 8, 500 mM NaCl, and 10% glycerol) and the reservoir solution (15% Jeffamine SD-2001, 0.1 M Na citrate, pH 5.4) at 22 °C. Crystals of DHRS9/NADH complex appeared in 1 to 2 weeks.

### Data collection

Prior to the data collection, suitable crystals were dipped for 30 s in a mother liquor solution with the addition of 10% glycerol as a cryoprotectant. Diffraction data were collected at 100 K at the NE-CAT beamline 24-ID-E at the Advance Photon Source. The images were processed using the XDS program suite (34) and scaled using Scala (44). Data collection and data processing statistics are given in Table 1.

### Crystal structure determination

The structure of DHRS9/NADH complex was determined by molecular replacement using the program MOLREP (44). A polyalanine model of a monomer of 17β-hydroxysteroid dehydrogenase (PDB accession code 6FFB; 29% sequence identity) was used as a search model. The positioned dimer was refined using the maximum likelihood refinement in REFMAC (44) with NCS restraints and one TLS group for each monomer. Coot (45) was used for model building throughout the refinement. The difference Fourier map revealed the presence of NADH in the active sites of the protein. The structure has been deposited with the Protein Data Bank with the accession code 9B2G. All structural figures were made using PyMOL software (DeLano Scientific, LLC (2005) The PyMOL Molecular Graphics System, Version 0.98.

### Statistical analysis

Statistical significance was determined using a two-tailed unpaired *t* test.

## Data availability

All data are contained within the manuscript.

## Supporting information

This article contains supporting information (46).

## Conflict of interest

The authors declare that they have no conflicts of interest with the contents of this article.
