# Supplementary material for: The effects of naturally occurring mutations on functionality of oxylipin metabolizing dehydrogenase reductase nine
Source: J Biol Chem. 2025 Sep 11;301(10):110704. doi: 10.1016/j.jbc.2025.110704 (PMC12538056; doi:10.1016/j.jbc.2025.110704)
Supplement: Supporting Tables and Figures [file mmc1.docx]

**SUPPORTING INFORMATION**

**The effects of naturally occurring mutations on functionality of oxylipin metabolizing dehydrogenase reductase 9**

Samuel E. Wirth,# Svetlana Pakhomova,$* Olga V. Belyaeva,# William E. Boeglin,Δ Alan R. Brash,Δ Marcia E. Newcomer,$ Natalia Y. Kedishvili,# Kirill M. Popov#*

**Table of Contents**

Table S1. Calculated probabilities of membrane localization of N-terminal amino acid residues of DHRS9.

Table S2. Secondary structure elements of DHRS9.

Figure S1. mRNA levels of S202L, D286H, and wild-type DHRS9 in HEK 293 cells.

Figure S2. Expression and purification of water soluble form of DHRS9.

Figure S3. Elution profile of water soluble form of DHRS9 protein from calibrated Superdex 75 10/300 GL column.

Figure S4. Catalytic activity of water soluble form of DHRS9 with various oxylipin substrates.

Figure S5. Primary and secondary structures of DHRS9 protein.

Figure S6. The schematic of DHRS9 active site.

Figure S7. The structural alignment of DHRS9 and 17*β*-HSD1 sequences.

Figure S8. Alignment of protein sequences of mouse and human DHRS9.

**Table S1.** **DeepTMHMM prediction of the membrane topology of DHRS9.** Calculated probabilities of membrane localization of N-terminal amino acid residues, which are likely to form transmembrane segment of mouse DHRS9.

| #AA | Membrane | Inside | Outside | Signal |
| --- | --- | --- | --- | --- |
| 0 M | 0 | 0 | 1 | 0 |
| 1 L | 0.77233 | 0 | 0.22781 | 0 |
| 2 F | 0.94913 | 0 | 0.051 | 0 |
| 3 W | 0.98418 | 0 | 0.01594 | 0 |
| 4 L | 0.99974 | 0 | 0.00037 | 0 |
| 5 L | 1 | 0 | 0 | 0 |
| 6 A | 1 | 0 | 0 | 0 |
| 7 L | 1 | 0 | 0 | 0 |
| 8 L | 1 | 0 | 0 | 0 |
| 9 F | 1 | 0 | 0 | 0 |
| 10 L | 1 | 0 | 0 | 0 |
| 11 C | 1 | 0 | 0 | 0 |
| 12 A | 1 | 2.00E-05 | 0 | 0 |
| 13 F | 0.99976 | 0.00033 | 0 | 0 |
| 14 L | 0.99529 | 0.00481 | 0 | 0 |
| 15 W | 0.93525 | 0.06484 | 0 | 0 |
| 16 N | 0.42358 | 0.57651 | 0 | 0 |

**Table S2. The elements of secondary structure of DHRS9 protein.**

| α Helices | Residue range | β Strands | Residue range |
| --- | --- | --- | --- |
| α1 | 41-52 | β1 | 30-36 |
| α2 | 63-72 | β2 | 55-60 |
| α3 | 87-101 | β3 | 78-82 |
| α4 | 128-155 | β4 | 107-111 |
| α5 | 176-198 | β5 | 157-165 |
| α6 | 216-227 | β6 | 200-207 |
| α7 | 231-238 | β7 | 279-283 |
| α8 | 242-251 |  |  |
| α9 | 262-272 |  |  |
| α10 | 285-288 |  |  |
| α11 | 292-295 |  |  |
| α12 | 299-308 |  |  |


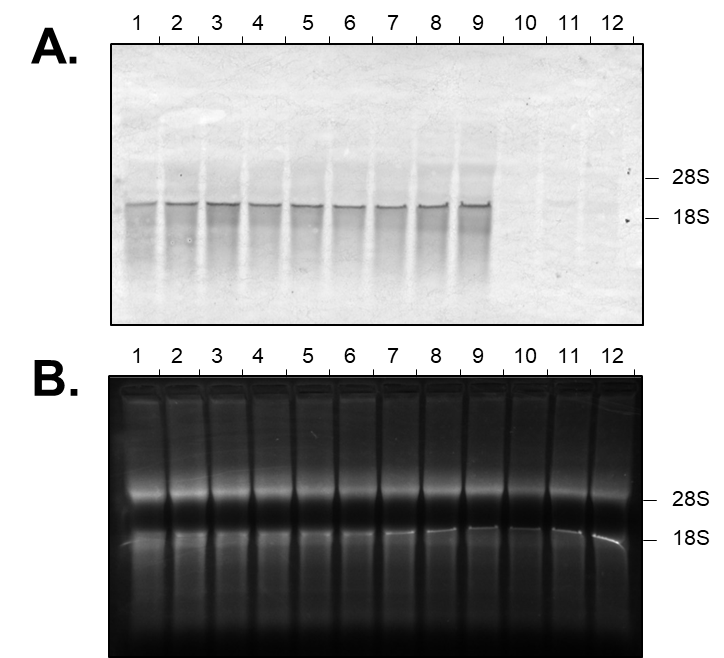


**Figure S1.** **mRNA levels of S202L, D286H, and wild-type DHRS9 in HEK 293 cells**. *A* – Northern blot analysis of S202L (*lines 1-3*), D286H (*lines 4-6*), and wild-type DHRS9 (*lines 7-9*) mRNAs in HEK 293 cells. RNA samples isolated from vector transfected HEK 293 cells used as a negative control (*lines 10-12*). *B* – Total RNA samples isolated from HEK 293 cells expressing of S202L (*lines 1-3*), D286H (*lines 4-6*), and wild-type DHRS9 (*lines 7-9*) separated on agarose gel. Total RNA samples isolated from vector transfected HEK293 cells used as a negative control (*lines 10-12*).


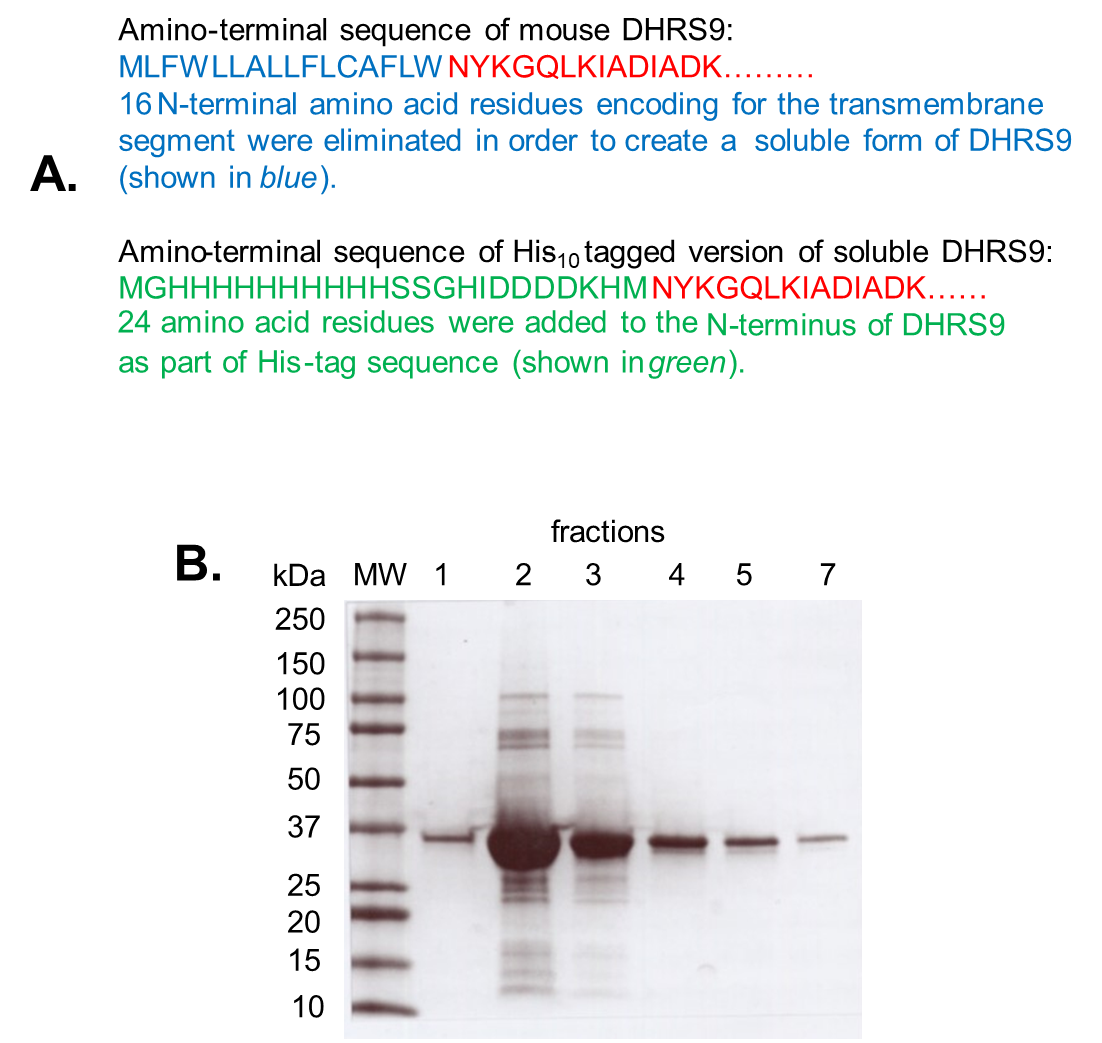


**Figure S2. Expression and purification of DHRS9**. *A* – The N-terminal amino acid sequences of wild-type enzyme and soluble DHRS9 construct. *B* – SDS/PAGE analysis of soluble DHRS9 protein purified by metal-affinity chromatography.


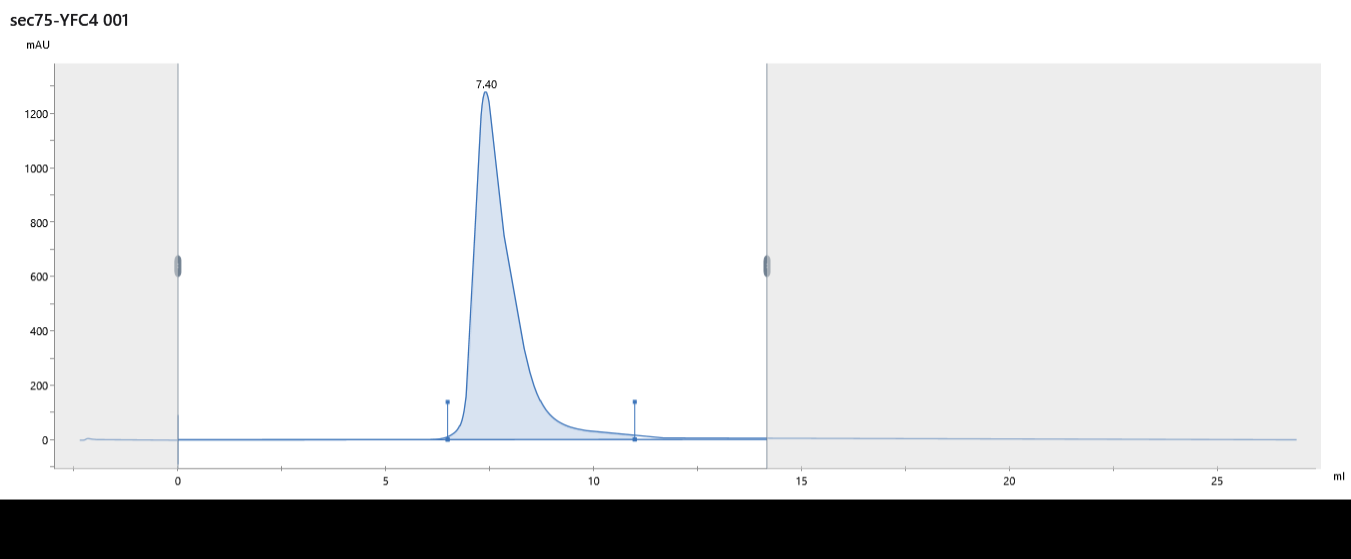


**Figure S3.** **Elution profile of soluble DHRS9 protein from calibrated Superdex 75 10/300 GL column.** Superdex column was equilibrated in 25 mM Tris pH 8, 0.5 M NaCl, 10% glycerol.

**
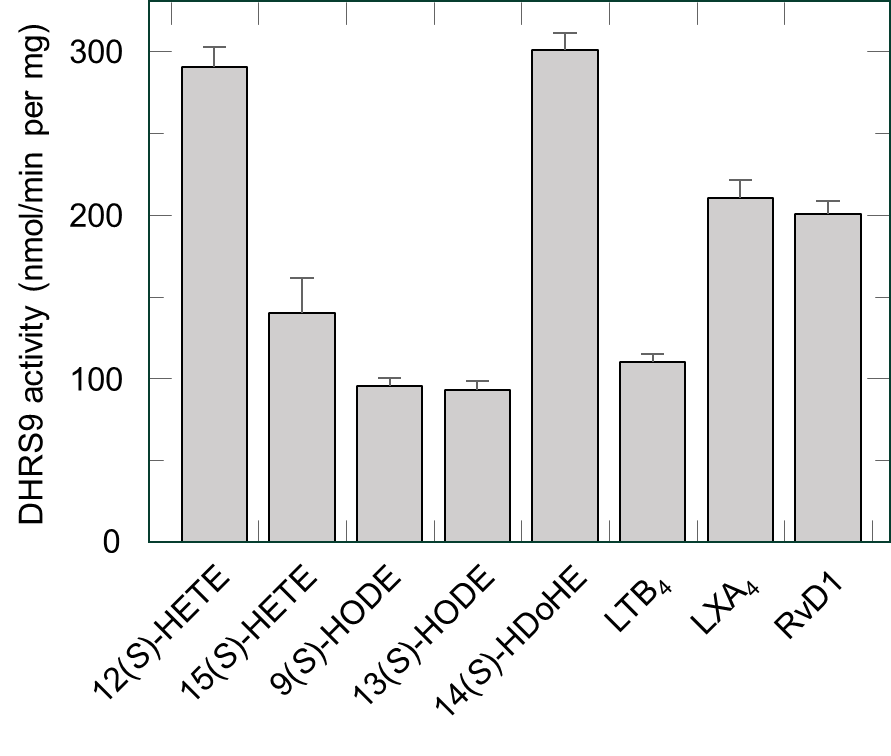
**

**Figure S4. Catalytic activity of water soluble form of DHRS9 with various oxylipin substrates.** Oxlylipin substrates were used at following concentrations: 12(*S*)-HETE 1.5 μM, 15(*S*)-HETE 2.0 μM, 9(*S*)-HODE 4.0 μM, 13(*S*)-HODE 1.5 μM, 14(*S*)-HDoHE 1.5 μM, LTB4 1.0 μM, LXA4 2.5 μM, and RvD1 5.5 μM. All measurements were carried out in the presence of 5 mM NAD^+^.

**β1 α1 β2 α2**

***sssssss hhhhhhhhhhhh ssssss hhhhhhhh***

**DHRS9 21 QLKIADIADKYVFITGCDTGFGNLAARTFDKKGFRVIAACLTESGSAALK**

**β3 α3 β4**

***hh sssss hhhhhhhhhhhhhhh sssss***

**DHRS9 71 AKTSERLHTVLLDVTDPENVKKTAQWVKSHVGEKGLWGLINNAGVLGVLA**

**α4 β5**

***hhhhhhhhhhhhhhhhhhhhhhhhhhhh sssssssss***

**DHRS9 121 PTDWLTVDDYREPIEVNLFGLINVTLNMLPLVKKARGRVINVSSIGGRLA**

**α5 β6**

***hhhhhhhhhhhhhhhhhhhhh ssssssss hhhhh***

**DHRS9 171 FGGGGYTPSKYAVEGFNDSLRRDMKAFGVHVSCIEPGLFKTELADPIKTT**

**α6 α7 α8 α9**

***hhhhhhh hhhhhhh hhhhhhhhhh hhhhhhhhh***

**DHRS9 221 EKKLAIWKHLSPDIKQQYGEGYIEKSLHRLKSNTSSVNLDLSLVVGCMDH**

**β7 α10 α11 α12**

***hh sssss hhhh hhhh hhhhhhhhhh***

**DHRS9 271 ALTSLFPKTRYIAGKDAKTFWIPLSHMPAVLQDFLLLKQKVELANPKAV**

**Figure S5. Primary and secondary structures of DHRS9 protein**.

**
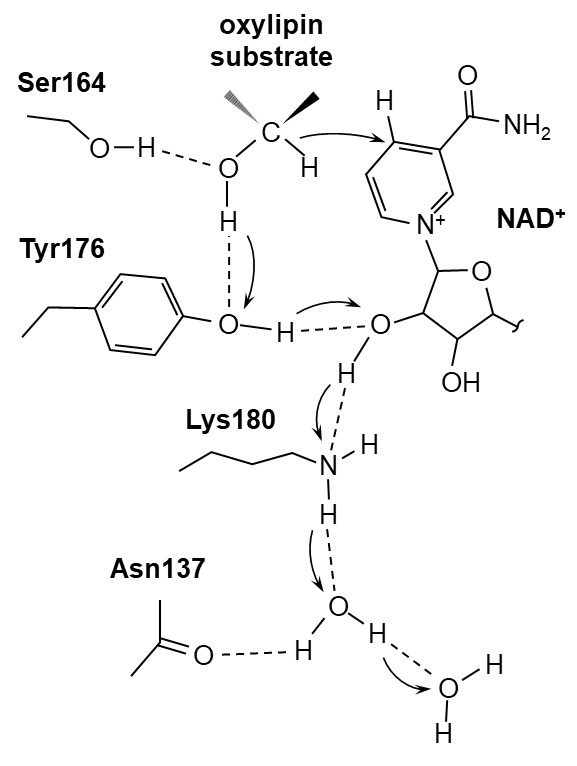
**

**Figure S6. The schematic of DHRS9 active site.** All building blocks characteristic of the catalytic site are present in DHRS9 structure. The side chains of Asn137, Ser164, Tyr176, and Lys180 can be clearly identified in the vicinity of nicotinamide moiety of nucleotide co-factor and retain the geometry characteristic of catalytic tetrad found in other short-chain dehydrogenases/reductases [29,30]. The hydroxyl group of Tyr176 is hydrogen bonded to the 2' OH hydroxyl group of ribosyl moiety of nucleotide co-factor . The catalytic Lys180 is hydrogen bonded to the same 2' OH hydroxyl group. In addition, Lys180 is also hydrogen bonded to a well-ordered water molecule. This water molecule is locked into place through the hydrogen bond interaction with the main chain of Asn137 and is further connected to the hydrogen bonding network of the water channel inside the protein fold. Thus, it appears that DHRS9 operates similar to other SDRs [29], i.e. the hydride ion is transferred to the 4-pro-S of the nicotinamide. The proton is moved through side chains of the active site tyrosine, lysine, the nicotinamide ribose hydroxyl and a conserved water molecule stabilized by the main-chain carbonyl of asparagine residue as part of "proton relay".

**β1 α1 β2**

***sssssss hhhhhhhhhhhh ssssss hhhhh***

**DHRS9 21 QLKIADIADKYVFITGCDTGFGNLAARTFDKKG---FRVIAACLTESGSA**

*** *.**** .*.* * . . *.* *. .**

**17*β*HSD 1 ARTVVLITGCSSGIGLHLAVRLASDPSQSFKVYATLRDLKTQG**

***ssssss hhhhhhhhhh ssssssss h***

**βA αB** **βB**

**α2 β3 α3 β4**

***hhhhh sssss hhhhhhhhhhhhhhh sssss***

**DHRS9 68 ALKAK------TSERLHTVLLDVTDPENVKKTAQWVKSHVGEKGLWGLIN**

*** . * *. *** *. .* . ..* * . *.**

**17*β*HSD 44 RLWEAARALACPPGSLETLQLDVRDSKSVAAARERV----TEGRVDVLVC**

***hhhhhhhhh sssssss hhhhhhhhh sssss***

**αC βC αD βD**

**α4 β5**

***hhhhhhhhhhhhhhhhhhhhhhhhhhhh ssss***

**DHRS9 112 NAGVLGVLAPTDWLTVDDYREPIEVNLFGLINVTLNMLPLVKKA-RGRVI**

*****. *.* . * * ..**. * . . ** .*. ***.**

**17*β*HSD 90 NAGLGL-LGPLEALGEDAVASVLDVNVVGTVRMLQAFLPDMKRRGSGRVL**

***hhhhhhhhhhh hhhhhhhhhhhhhhhh ssss***

**αE' αE βE**

**α5 β6**

***sssss hhhhhhhhhhhhhhhhhhhhh ssssssss***

**DHRS9 161 NVSSIGGRLAFGGG-GYTPSKYAVEGFNDSLRRDMKAFGVHVSCIEPGLF**

**.*.** ... * .**.*.**. .** . .****.* ** ***

**17*β*HSD 139 VTGSVGGLMGLPFNDVYCASKFALEGLCESLAVLLLPFGVHLSLIECGPV**

**sssss *hhhhhhhhhhhhhhhhhhhhhhh* *sssssss***

**αF** **βF**

**α6 α7 α8**

***hhhhhhhhhhhh hhhhhhh hhhhhhhhhh***

**DHRS9 210 KTELADPIKTTEKKLAIWKHLSPDIKQQYGEGYIEKSLHRLKSNTSS-VN**

*** . . . . . ***

**17*β*HSD 189 HTAFMEK-VLGSPEEVLDR------TDIHTFHRFYQYLAHSKQVFREAAQ**

***hhhhh hhhhhhhhhhhhhhhhhhhhh***

**αG" αG'**

**α9 β7 α10 α11 α12**

***hhhhhhhhhhh sssss hhhh hhhh hhhhhhhh***

**DHRS9 259 LDLSLVVGCMDHALTSLFPKTRYIAGKDAKTFW--IPLSHMPAVLQDFLL**

**. * ** . * **.. . . .**

**17*β*HSD 232 -NPEEVAEVFLTALRAPKPTLRYFTTERFLPLLRMRLDDPSGS-NYVTAM**

***hhhhhhhhhhhhh sssss hhhh hhhhhh***

**αG βG αH' αH**

***hh***

**DHRS9 307 LKQKVELANPKAV**

**..**

**17*β*HSD 280 HREVFG**

***hhhhh***

**Figure S7. The structural alignment of DHRS9 and 17*β*-HSD type 1 sequences.** Alignment is based on the superimposition of DHRS9 and 17*β*-HSD type 1 structures shown in Fig. 4B.

**mouse DHRS9 QLKIADIADKYVFITGCDTGFGNLAARTFDKKGFRVIAACLTESGSAALKAKTSERLHTV**

**human DHRS9 KLKIEDITDKYIFITGCDSGFGNLAARTFDKKGFHVIAACLTESGSTALKAETSERLRTV**

**:*** **:***:******:***************:***********:****:*****:****

**mouse DHRS9 LLDVTDPENVKKTAQWVKSHVGEKGLWGLINNAGVLGVLAPTDWLTVDDYREPIEVNLFG**

**human DHRS9 LLDVTDPENVKRTAQWVKNQVGEKGLWGLINNAGVPGVLAPTDWLTLEDYREPIEVNLFG**

*************:******.:*************** **********::**************

**mouse DHRS9 LINVTLNMLPLVKKARGRVINVSSIGGRLAFGGGGYTPSKYAVEGFNDSLRRDMKAFGVH**

**human DHRS9 LISVTLNMLPLVKKAQGRVINVSSVGGRLAIVGGGYTPSKYAVEGFNDSLRRDMKAFGVH**

****.************:********:*****: ******************************

**mouse DHRS9 VSCIEPGLFKTELADPIKTTEKKLAIWKHLSPDIKQQYGEGYIEKSLHRLKSNTSSVNLD**

**human DHRS9 VSCIEPGLFKTNLADPVKVIEKKLAIWEQLSPDIKQQYGEGYIEKSLDKLKGNKSYVNMD**

*************:****:*. *******::******************.:**.*.* **:***

**mouse DHRS9 LSLVVGCMDHALTSLFPKTRYIAGKDAKTFWIPLSHMPAVLQDFLLLKQKVELANPKAV**

**human DHRS9 LSPVVECMDHALTSLFPKTHYAAGKDAKIFWIPLSHMPAALQDFLLLKQKAELANPKAV**

**** ** *************:* ****** **********.**********.**********

**Figure S8.** Alignment of protein sequences of mouse and human DHRS9. Protein sequences were aligned using Clustal X software. In the alignment, an asterisk (*) signifies a fully conserved position; a colon (:) indicates conserved substitutions; and a period (.) represents semi-conserved substitutions.
